# Micro-CT and Microscopy Study of Internal and Marginal Gap to Tooth Surface of Crenelated versus Conventional Dental Indirect Veneers

**DOI:** 10.3390/medicina57080772

**Published:** 2021-07-29

**Authors:** Alexandra-Cristina Măroiu, Cosmin Sinescu, Virgil-Florin Duma, Florin Topală, Anca Jivănescu, Paul Mircea Popovici, Anca Tudor, Mihai Romînu

**Affiliations:** 1 Research Center in Dental Medicine Using Conventional and Alternative Technologies, School of Dental Medicine, “Victor Babes” University of Medicine and Pharmacy of Timisoara, 9 Revolutiei 1989 Ave., 300070 Timisoara, Romania; maroiualexandra@gmail.com (A.-C.M.); minosinescu@gmail.com (C.S.); popovici.paul@umft.ro (P.M.P.); anca.ancutza@gmail.com (A.T.); mrominu@hotmail.com (M.R.); 2 3OM Optomechatronics Group, Faculty of Engineering, “Aurel Vlaicu” University of Arad, 2 Elena Dragoi Str., 310177 Arad, Romania; 3 Doctoral School, Polytechnic University of Timisoara, 1 Mihai Viteazu Ave., 300222 Timisoara, Romania; 4 School of Dental Medicine, “Victor Babes” University of Medicine and Pharmacy of Timisoara, 2A Eftimie Murgu Place, 300070 Timisoara, Romania; florin.topala@gmail.com (F.T.); ajivanescu@yahoo.com (A.J.)

**Keywords:** dental veneers, crenelated veneers, lithia disilicate, computer-aided design, computer-aided manufacturing, dental cement, dental marginal adaptation, microscopy, micro-computed tomography (CT), patient-specific modeling

## Abstract

*Background and Objectives*: Ceramic veneers represent the most appropriate treatment option for minimally invasive aesthetic rehabilitation. For long-term clinical success, the accurate marginal and internal adaptation of dental restorations are of paramount importance. The aim of this in vitro study is to assess the effect of a novel (patented) design of veneers compared to conventional ones on their marginal and internal gap to the prepared tooth surface. *Materials and Methods*: Twenty-four lithium disilicate ceramic veneers are obtained using Computer-Aided-Design (CAD) and then milled using Computer-Aided-Manufacturing (CAM). The samples are divided into two groups: 12 conventional (CO) veneers (i.e., with a linear marginal contour) and 12 crenelated (CR) veneers, the latter with the novel sinusoidal marginal design. All samples are bonded to frontal teeth, and the adhesive interfaces are analyzed using two methods, optical microscopy and micro-Computed Tomography (CT): the former for the accuracy of the marginal gap and the latter for the internal gap (as well as for the homogeneity of the luting cement) of ceramic veneers. *Results*: STATA and one-way ANOVA tests reveal significant differences between CO and CR veneers: (i) the marginal gap is smaller for CR (64 μm) than for CO veneers (236 μm); (ii) the internal adaptation is better for CR veneers: for a cement width of up to 120 μm, the covered surface for the CR group is 81.5%, while for the CO group it is 64.5%; (iii) the mean of the porosities within the cement is not significantly different (3.4·10^6^ μm^3^ for CO and 3.9·10^6^ μm^3^ for CR veneers), with a higher standard deviation for the CO group. Analytical modeling is achieved for internal gaps using the micro-CT results. The characteristic functions obtained allow us to compare the volume of luting cement for the two types of veneers. *Conclusion*: The novel veneers design produces an improvement in the marginal and internal adaptation of the restorations to the prepared tooth surface. Thus, it provides favorable premises for better clinical performances.

## 1. Introduction

Aesthetic dentistry has become one of the most dynamic fields of modern dentistry. The concepts of beauty and nature meet in the option of minimally invasive treatment, and ceramic dental veneers play a key role in this context. Such prosthetic restorations are deemed to augment both the functional and aesthetic appearance of worn, colorless, or mispositioned teeth by performing a conservative preparation of the dental structure [1].

The history of dental veneers had begun in the 1930s when Charles Pincus invented them to enhance the beauty of American actors’ smiles [2]. The introduction of etching in 1959 by Dr. Michael Buonocore aimed to follow a line of investigation of bonding ceramic veneers to etched enamel [3]. Since then, numerous experimental studies have been conducted with the scope to improve both their mechanical and cosmetic properties. Research directions have been focused on materials and technological methods to fabricate veneers, luting materials, and protocols of the veneers to the dental surface, as well as on different tooth preparations for prospective veneers [4,5,6,7].

Regarding the latter direction of research, four basic designs for teeth preparation have been described in the literature: window, feather, bevel, and incisal overlap [6]. They all have *a linear marginal contour* of the preparation in common, as described in the following section. This design has the drawbacks of a small contact surface with the adjacent enamel and of a large amount of sound tooth removal [7,8]. In consequence, many cases of clinical failure have been reported due to the debonding of veneers from dental support [9]. Furthermore, veneer preparation in dentin adversely affects survival [9]; therefore, in our work, all veneers are bonded to enamel.

Even if the adhesive properties of dental materials to the tooth structure have been considerably enhanced by current adhesives [5], we consider that practitioners should not rely exclusively on them. Instead, as an alternative approach, one may explore other possible designs of tooth preparation. They should be highly conservative but providing the best possible adhesive and mechanical properties.

The importance of the thickness/width of the marginal and internal gap of veneers for the clinical success of ceramic restorations has been emphasized in several clinical trials [10,11,12,13,14,15,16]. The marginal gap (i.e., the perpendicular distance from the peripheral margins of the restoration to its finish line) should be small enough to prevent ingress of saliva and/or lactic acid, which is the byproduct of bacterial metabolism. Marginal adaptation is one of the basic factors in the success of restorations [17,18]; a poor one may lead to cement dissolution, marginal discoloration or staining, microleakage, secondary cavities, restoration debonding, and, eventually, restoration fracture. Therefore, it is important to minimize marginal gaps to decrease the incidence of associated complications [19]. Moreover, it has been demonstrated that increased marginal discrepancy values reduce the fracture resistance of the veneering ceramic [20]. The close proximity between the margin of restorations and the tooth structure protects the adhesive resin cement from excessive exposure to the oral cavity, which would eventually lead to the slow process of gradual decrease of its chemical, physical, and mechanical properties. These may also produce microleakage, recurrent decay, discoloration of the tooth structure, and fracture of the veneers. On the other hand, the internal marginal gap is a direct measure of the cement film thickness underneath the restoration and is significantly influenced by the accuracy of the fabrication process [21]. A large cement thickness under the ceramic restoration especially causes mechanical failure because of the limited shear strength [22]. Although various studies showed controversial results concerning the clinically acceptable gap values, most of them agreed that a marginal and internal discrepancy between 100 and 120 μm appears to be in the range of clinical acceptance [23,24,25]. Therefore, this limit is considered in the present study as well.

Taking the above aspects into consideration, the general scope of our direction of work has been to develop *a novel, sinusoidal marginal design* of the preparation. This new type of dental veneer, which we called *“crenelated*” (CR), is the subject of a patent of our group [26], as it has not been previously developed, to the best of our knowledge. In this respect, this could be considered a pilot study. We introduced the proposed design in a preliminary work that investigated the adhesive force between the novel veneers and resin tooth models [27], exploring the capability of this novel solution to augment the adhesion, therefore, the mechanical properties of veneers. This may be considered as an indirect indication of an as tight as possible interface between them and their dental support.

The aim of the present study is to assess the influence that such a CR design has over the marginal and internal adaptation of the veneers in comparison to the conventional (CO) design. An in vitro study must be carried out to complete this comparison between the CO and the CR veneers. Two imaging methods are used for the proposed assessment: optical microscopy for the marginal adaptation and micro-CT for a volumetric/three-dimensional (3D) investigation of internal gaps. The latter must also be used for a porosity analysis, which is necessary to assess avoiding leakages, micro-infiltration, and fissures within the cement layer. Such micro-CT investigations come in the context of their use for a range of studies, including dental adhesives [28] or bone regeneration [29,30], coupled with other investigation methods, for example, microscopy [28] or optical coherence tomography (OCT) [31].

The first study hypothesis is that the novel CR veneers provide a better marginal and internal adaptation in comparison to CO veneers. The second study hypothesis is that they produce a better homogeneity of the dental cement.

## 2. Materials and Methods

The study was conducted according to the guidelines of the Declaration of Helsinki. It was approved by the CECS nr. 5/19.01.2021 of the Ethical Committee of the “Victor Babes” University of Medicine and Pharmacy of Timisoara, Romania.

### 2.1. Tooth Surface Preparation

Twenty-four human extracted teeth, displaying no decays or resin fillings, are prepared on their vestibular face. Thus, 24 veneers are obtained. Considering the type of tooth preparation, the teeth are divided into two experimental groups, each group consisting of four upper right lateral incisors, four upper left central incisors, and four upper left canines: Group 1, with 12 CO veneers and Group 2, with 12 CR veneers [26]. The teeth are stored in distilled water, at 25 °C, during the entire investigation process. To standardize teeth preparations, they have been processed only by one author using a 4× magnification system (Univet, Italy). The veneers are manufactured from lithium disilicate ceramic (IPS e.max, Ivoclar Vivadent, Liechtenstein).

**Figure 1:**
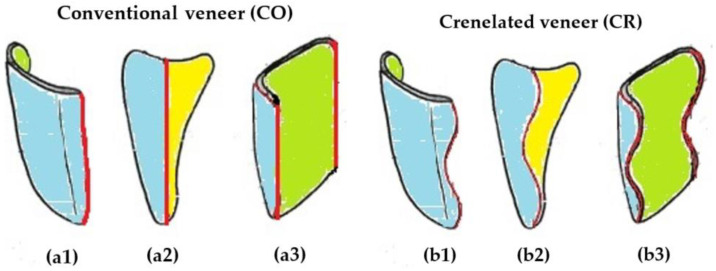
The two veneers designs prepared for the study (to be compared with regard to their adhesive properties): (**a**) conventional (CO)-with linear margins and (**b**) crenelated (CR)-with sinusoidal margins, the latter patented by our group [26]. Notations and color codes of the three views of each type of veneer: (**1**) vestibular view of the external face (blue); (**2**) proximal view displaying the peripheral intrications between the marginal contour of the veneer (blue) and the adjacent prepared tooth structure (yellow); (**3**) inner surface of the veneer (green).

For all the 12 samples of Group 1, the CO tooth preparation for dental veneers is performed by reducing the facial face by 0.5–0.8 mm and the incisal margin by 1 mm. The proximal contour is linear, with its limits positioned just before the interdental contact (Figure 1a).

For all the 12 samples of Group 2, the novel CR tooth preparation consists of three sinusoidal marginal lines which outline the contour of the facial dental veneer (Figure 1b). The proximal limits are positioned just before the interdental contact. The height of the crenelated lines is correlated with the type of the tooth, namely: 2–2.5 mm for lateral and inferior central incisors and 2.5–3 mm for central upper incisors and canines. The depth of the sinusoidal proximal margins decreases progressively from 0.6 to 0.8 mm in the gingival third, towards 0.4–0.6 mm in the middle third, and towards 0.3–0.4 mm in the incisal third. The facial face of the tooth is reduced by 0.5–0.8 mm, and the incisal margin by 1 mm to provide an aesthetic final restoration.

These CR dental veneers have a specific design that fits with the preparation of the tooth: three sinusoidal proximal margins with different heights and depths, 0.5–0.8 mm facial thickness, and 1 mm incisal thickness [26,27]. Samples of both groups are characterized by a butt joint finish line situated at the incisal margin and a chamfer finish line along the cervical and proximal contour.

The preparation time for a CR veneer is similar to a CO one, as the sequences of the tooth reduction and finishing protocols are alike. Figure 1 displays single teeth in order to outline the different marginal contour designs for CR and CO veneers. The design of the incisal and vestibular preparation is the same when adjacent teeth are present. The only aspect to discuss is whether to extend the finishing line anterior or posterior to the interproximal contact. If it is necessary to change the initial tooth color or form or to close diastemas or triangular black spaces between teeth, then the marginal finishing line extends beyond the interproximal contact. If none of these conditions are present, then the finishing line of the tooth preparation extends anterior to the interproximal contact with the adjacent teeth.

### 2.2. Manufacturing of the Dental Veneers

**Figure 2:**
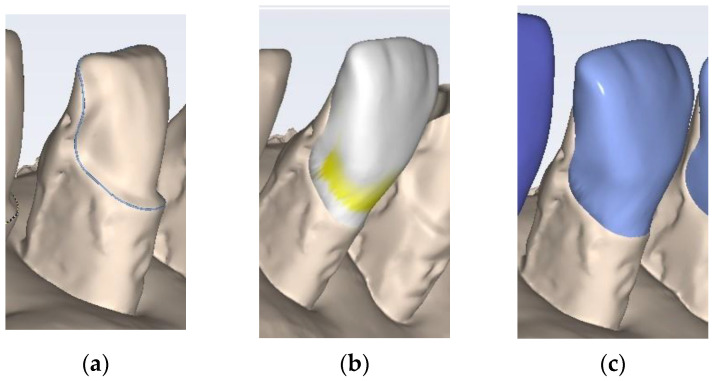
The main steps of elaborating the CR veneers using the Planmeca Romexis Software: (**a**) tracing the outline of the preparation; (**b**) establishing the design of the veneer; (**c**) commanding the milling process.

The designing and manufacturing processes of both types of veneers (i.e., CO and CR) are based on Computer-Aided-Design (CAD) and on Computer-Aided-Manufacturing (CAM). The Planmeca FIT^®^ system (Planmeca OY, Helsinki, Finland) is used, namely, the chairside CAD/CAM system that combines the entire restorative workflow, from scanning to designing and milling (Figure 2). This allows clinics to produce restorations in a single patient’s visit. Thus, the Planmeca PlanScan Intraoral Scanner (Planmeca OY, Helsinki, Finland), which is part of the Planmeca Romexis all-in-one dental software platform, is used to scan the tooth preparations. Consequently, digital impressions of these preparations are obtained by capturing accurate 3D images to generate appropriate virtual models. The kit of diamond burs used for the tooth preparation design is coarse-grained cylindric burs (0.8 mm and 1 mm in diameter), fine-grained cylindric burs (0.8 mm and 1 mm in diameter), rounded-tip Arkansas stone burs, and super-fine polishing discs (Komet Dental, Germany). The tooth preparation sequence consists of: reduction of the incisal margin and of the vestibular face using coarse-grained burs; preparation of the linear or sinusoidal marginal line (using the same burs); finishing and polishing the dental surface with fine-grained cylindric burs, Arkansas stone burs, and super-fine polishing discs.

The next step is to outline the contour of the preparation, which coincides with the marginal contour of the final dental veneers. The design of CO and CR veneers is produced considering both the marginal contour and the anatomical morphology of the natural teeth to be restored. The preparation of the incisal edge is the same for CO and CR veneers, namely, butt-joint preparation, performed by a perpendicular movement of the bur onto the incisal margin. This addresses the issue described by previous studies, i.e., higher internal gaps in the occlusal/incisal area [32].

The final step of the manufacturing is the milling process, performed with the Planmeca PlanMill 40S machine (Planmeca OY, Helsinki, Finland), which may work with different indications and in-house materials. The selected milling parameters are: two spindles for enhanced milling speeds, 4 axis, 80 krpm milling speed, and wet processing. The average milling time (for ceramic, hybrid ceramic, and zirconium restorations) is 8–10 min. The lithium disilicate glass-ceramic blocks used for the milling process, namely, IPS e.max CAD HT Monolithic (Ivoclar Vivadent, Schaan, Lichtenstein), streamline the fabrication of full-contour restorations, with durability, proven clinical properties, as well as excellent esthetics and high strength of 530 MPa. The crystallization oven is PROGRAMAT P510 (Ivoclar Vivadent, Schaan, Lichtenstein), and the utilized program is P161. All crystallization and glazing steps are performed according to the manufacturer’s indications.

All 24 ceramic veneers are luted to the prepared tooth surfaces (i.e., to enamel) using the Variolink Esthetic Light Cure system (Ivoclar Vivadent, Schaan, Lichtenstein), after a rigorous surface conditioning of teeth and restorations, according to the manufacturer’s indications. Thus, the adhesive interface between the dental support, cement, and inner surface of dental veneers is obtained to be investigated in the following. There is no cement-thickness preset in the software. The luting cement layer is measured using the micro-CT software tools after the luting procedure is performed. No transfer keys are used during the luting procedure because the finishing lines of the tooth preparation are precise; therefore, the positioning of the veneers is guided by the marginal contour itself. Moreover, the sinusoidal design of the novel preparation facilitates a more accurate placement of the veneers because of its marginal micro-retentions.

The internal fit of restoration also depends on the cementation force [33]. The ceramic restorations are luted in this work by hand, with controlled pressure and precision that depends on the dentist’s experience, as in clinical practice. The standardization of the luting procedure in this study is assured by having a single operator who performs the same luting steps for all samples.

### 2.3. Optical Microscopy

The most common investigation method available to practitioners, i.e., optical microscopy, is used to evaluate the interfaces between the dental veneers and the subjacent enamel to measure the marginal gap filled with residual luting cement. This gap (i.e., the dental cement thickness/width) has been defined as the maximum distance between the finish line of the underlying prepared tooth and the margin of ceramic dental veneer. This width of the marginal residual luting cement must be studied because it is related to the microleakage potential, as pointed out in the Introduction.

All microscopy measurements are performed by engineers specialized in optical metrology (Top Metrology Laboratory, Bucharest, Romania). The same evaluators perform both the measurements and the data analysis.

**Figure 3:**
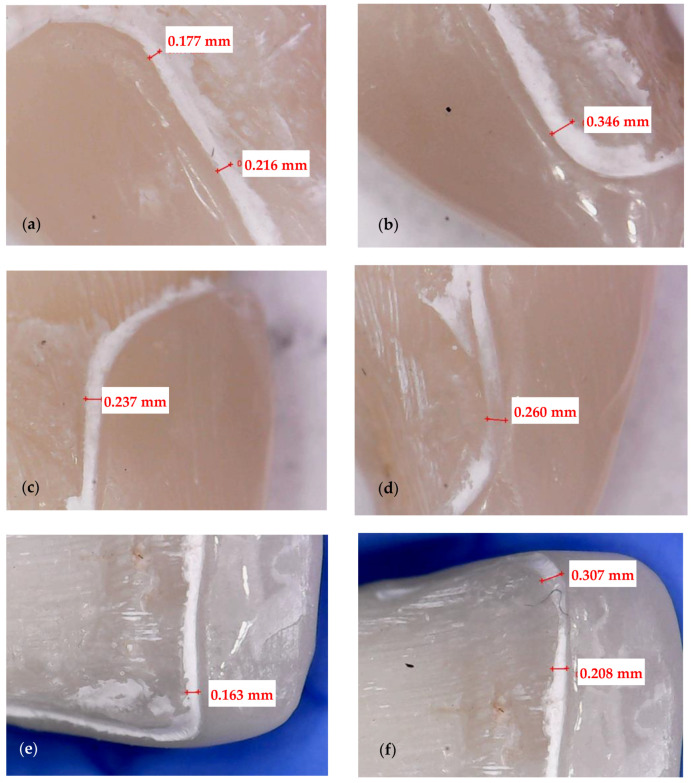
Conventional (CO) veneer on an upper right lateral incisor. Measurements of the marginal gaps along: (**a**) the mesial surface (cervical and middle third); (**b**) the mesial surface (incisal third); (**c**) the distal surface (cervical third); (**d**) the distal surface (incisal third); (**e**) the incisal surface (distal third); (**f**) the incisal surface (distal third). The white band provides the thickness/width of the luting cement.

**Figure 4:**
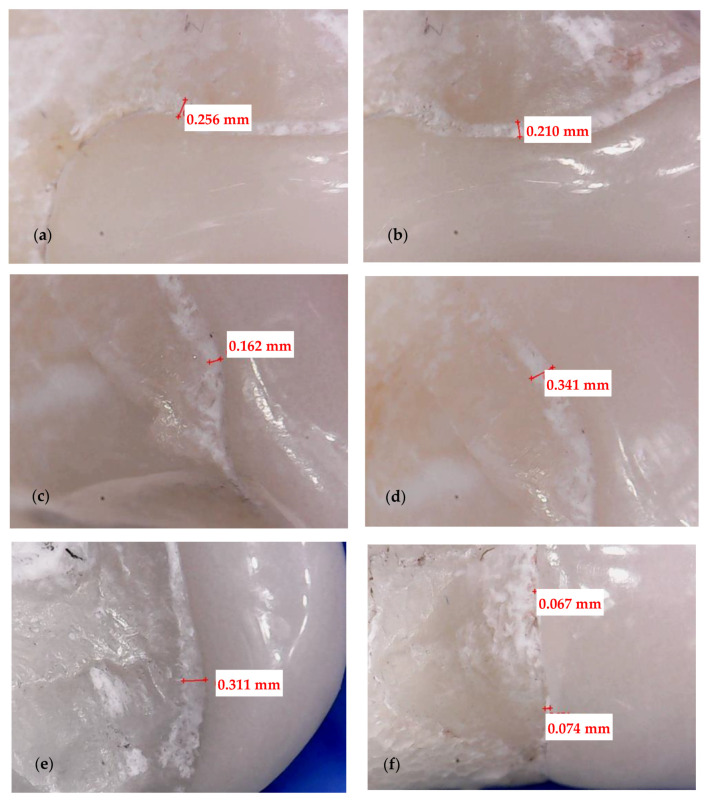
Crenelated (CR) veneer on an upper left canine. Measurements of the marginal gaps along: (**a**) the distal surface (cervical third); (**b**) the distal surface (central third); (**c**) the mesial surface (incisal third); (**d**) the mesial surface (central third); (**e**) the incisal surface (distal third); (**f**) the cervical surface. The white band provides the thickness/width of the luting cement.

Four surfaces are considered for each tooth: cervical, incisal, mesial, and distal (Figure 3 and Figure 4). Each corresponding interface is divided into three segments, thus generating the following sites of interest: incisal surface—mesial third, middle third, and distal third; cervical surface—mesial third, middle third, and distal third; mesial surface—incisal third, middle third, and cervical third; distal surface—incisal third, middle third, and cervical third. The marginal gaps are measured in five points for each of these segments. Thus, 15 points of measurement are considered for each surface and 60 points in total along the adhesive interfaces for each tooth.

Both experimental groups (CO and CR) are analyzed with the digital microscope AmScope UWT (AmScope, Irvine, CA, USA) with the magnification set at 50×. Its software, CoolingTech (Informer Technologies, Inc., Roseau, Dominica), allows to capture pictures of each segment (where the luting cement is visible) and to measure the width of the adhesive material. The resolution of the triggered images is set to 640 × 480. In the imaging software, the measured parameters expressed in pixels are converted through the system calibration into real absolute units (by comparing an object of known size with a software-generated scale). Shots of the margins are taken for each veneer. Marginal gaps are measured as the vertical distance between the finish line of the preparation and the veneer margin. The measurements are done along the peripheral circumference for all the veneer margins (i.e., mesial, distal, cervical, and incisal).

Measurements in each point are repeated five times, and a mean value is obtained. The obtained data are collected, and further on, subjected to statistical analysis. This experimental protocol is applied to all 12 CO and to all 12 CR veneers. A representative specimen of each group is presented to describe the investigation process: a CO veneer on an upper right lateral incisor (Figure 3) and a CR veneer on an upper left canine (Figure 4).

The sites identified as potential marginal gaps are further investigated with micro-CT to determine if the thickness of the marginal residual luting cement was triggered by the misfit of the dental veneers or by clinical errors that occurred during the luting process.

### 2.4. Micro-CT Investigations

Micro-CT is a non-destructive tool that comprehensively investigates 3D details of the scanned objects [34]. Micro-CT investigations are used in this study to determine the internal and marginal adaptation of the CO and CR veneers by measuring the thickness/width of the luting cement localized at the adhesive interface. Moreover, these investigations allow the identification of the number and dimensions of porosities within the luting cement, as presented in the next subsection. Thus, a correlation between the two types of tooth preparation and the homogeneity of the luting cement can be triggered. This is important for the long-term clinical success of the prosthetic treatment.

Each tooth included in the study is positioned in supports designed for easy handling during scanning. A Nikon XTH 225 ST system (Nikon Metrology, Inc., Brighton, USA) is used. This CT system, suited for a wide range of materials and sample sizes, is operated with the following parameters: 145 kV tube voltage, 145 μA tube current, and 1440 angular projections acquired over a rotation of 360°, with an exposure time of 1.42 s. The software used is Inspect-X (Nikon Metrology, Inc., Brighton, MA, USA), and the scanning session takes 70 min per tooth. The images obtained are imported into Volume Graphics Studio 3.2 Max (Volume Graphics Gmbh, Heidelberg, Germany), and then 3D/volumetric samples reconstruction and analysis are completed. As in the case of the microscopy assessments, the same evaluators perform both the measurements and the data analysis.

This analysis of the adhesive interfaces is conducted on both the marginal contour and on the vestibular face of the two types of tooth preparations. It has two purposes: to measure the thickness/width of the luting cement (i.e., a *wall thickness analysis*) and to evaluate the number and dimension of the porosities included in the luting cement (i.e., *porosities analysis*). As a remark, no standardization in the measurement points of marginal gaps is made for a direct comparison between microscopy and micro-CT, as the former can offer only measurements in certain points, while the latter allows for a 3D view and assessment of the entire cement layer. Additionally, no identification of the measuring points using microscopy can be made precisely on the 3D micro-CT images. However, the statistical analysis of both sets of results (i.e., obtained with both methods) can provide a relevant comparison between the two methods that may thus validate each other.

**Figure 5:**
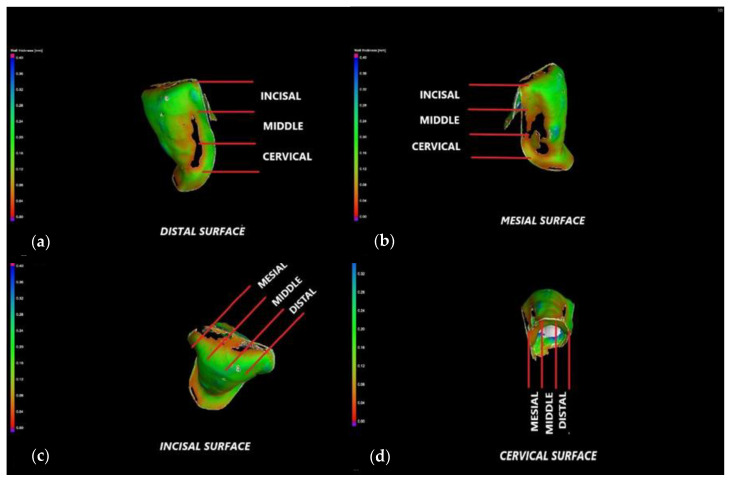
The marginal contour of the CO tooth preparation (upper right lateral incisor), displaying the thickness/width *w* of the luting cement, which is investigated on each surface, divided into three thirds: (**a**) distal: incisal/middle/cervical; (**b**) mesial: incisal/middle/cervical; (**c**) incisal: mesial/middle/distal.; (**d**) cervical: mesial/middle/distal.

**Figure 6:**
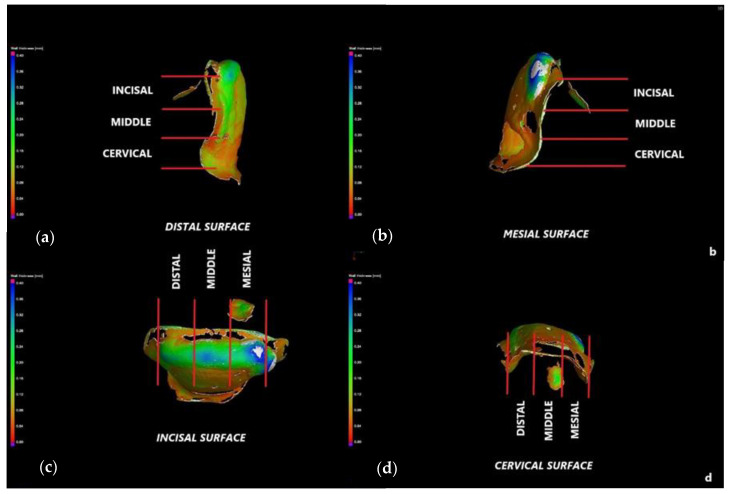
The marginal contour of the CR tooth preparation (upper left canine), displaying the thickness/width *w* of the luting cement, which is investigated on each surface, divided into three thirds: (**a**) distal: incisal/middle/cervical; (**b**) mesial: incisal/middle/cervical; (**c**) incisal: mesial/middle/distal.; (**d**) cervical: mesial/middle/distal.

The marginal contour of each of the 24 specimens is defined by the same four surfaces described in Section 2.3 and presented in Figure 3 and Figure 4: mesial, distal, incisal, and cervical. Each surface is divided into three segments, thus generating the following sites of interest: incisal surface—mesial, middle, and distal third; cervical surface—mesial, middle, and distal third; mesial surface—incisal, middle, and cervical third; distal surface—incisal, middle, and cervical third. The cement thickness/width is measured in 5 points of each segment, generating 15 values per surface, and a total of 60 values for the entire marginal contour. To validate the accuracy of the results obtained with optical microscopy, Figure 5 and Figure 6 present the way micro-CT investigations are performed in detail for the same two specimens previously displayed in Figure 3 and Figure 4, respectively—namely, a CO veneer on an upper right lateral incisor and a CR veneer on an upper left canine.

**Figure 7:**
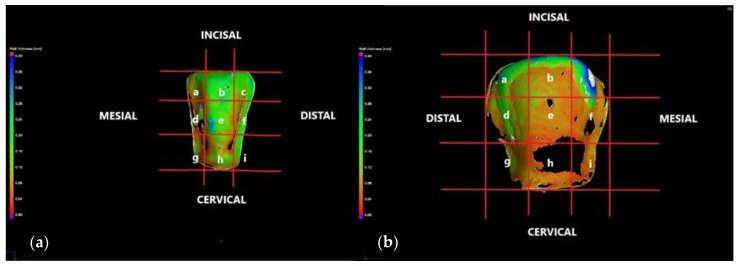
The vestibular face of (**a**) the CO tooth preparation, displaying the thickness/width *w* of the luting cement on an upper right lateral incisor and of the (**b**) CR tooth preparation on an upper left canine, both divided into nine quadrants of measurement: a, b, c, d, e, f, g, h, i.

The vestibular face of each specimen is divided in three horizontal regions—incisal/middle/cervical, and each region is split into three segments. Therefore, each CO and CR tooth preparation had nine quadrants of measurement, situated along the vestibular face and beneath the corresponding ceramic veneer, as shown in Figure 7.

**Figure 8:**
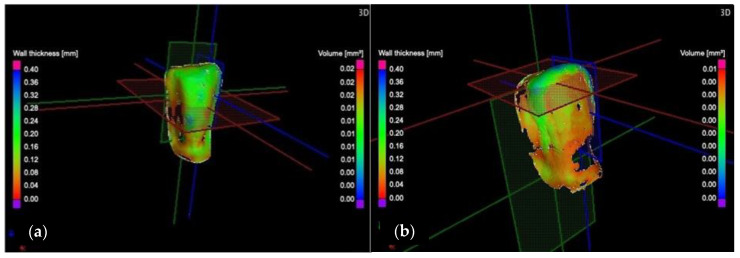
3D analysis of the wall thickness/width *w* of samples: (**a**) CO and (**b**) CR tooth preparation, displaying the dimension scale (in a color code) on the left side.

The samples are investigated in 3D, according to the three planes marked in Figure 8: X (red marked), the transversal plane; Y (green marked), the frontal plane; Z (blue marked), the sagittal plane.

**Figure 9:**
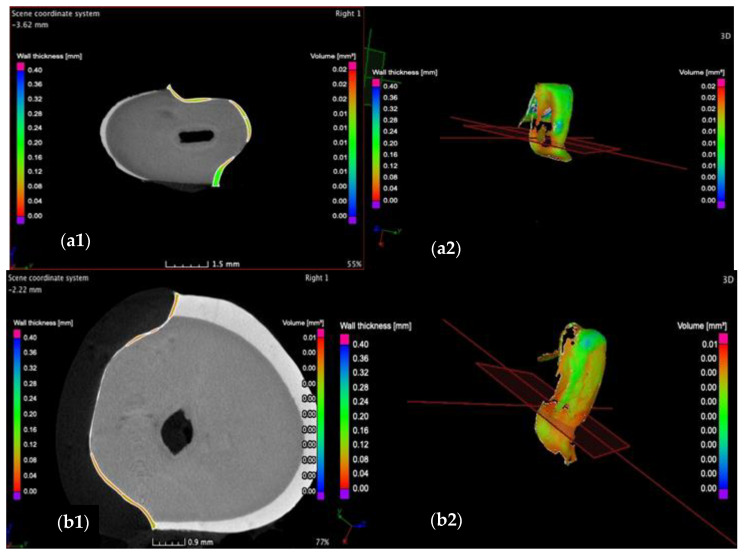
Investigation of the cervical region of the vestibular face of (**a**) the CO and of (**b**) the CR tooth preparation in the transversal plane, displaying a maximum cement width *w* of 200 μm for the CO and 100 μm for the CR sample. Moreover, the latter features a more uniform dispersion of the luting cement compared to the former. Notations: (**1**) section and (**2**) 3D reconstruction.

**Figure 10:**
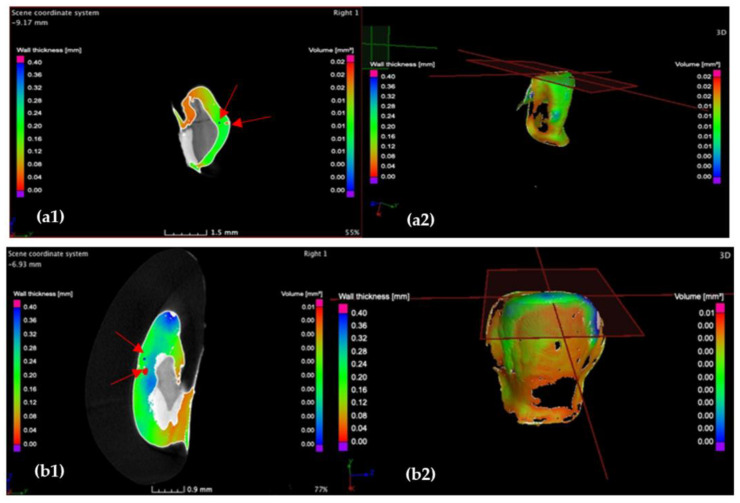
Investigation of the incisal marginal contour of the CO (**a**) and CR tooth preparation (**b**) in the transversal plane, displaying a maximum cement width *w* of 240 μm for the CO and 350 μm for the CR sample. Moreover, porosities (red arrows) are identified at the incisal margins of both preparations. Notations: (**1**) section and (**2**) 3D reconstruction.

To determine the marginal and internal adaptation of the ceramic veneers, the *wall thickness/width analysis* is carried out. This provides data about the width *w* of the luting cement and its dispersion along the adhesive interfaces. This width is displayed on the left side of each picture as a gradient color scale from red to blue, ranging from 0 to 400 μm. The investigation starts by performing transversal sections, moving the axis from the cervical surface to the incisal surface. Thus, images of the marginal contours and internal adhesive interfaces are captured. Two sections from these investigations are shown as examples in Figure 9 and Figure 10.

**Figure 11:**
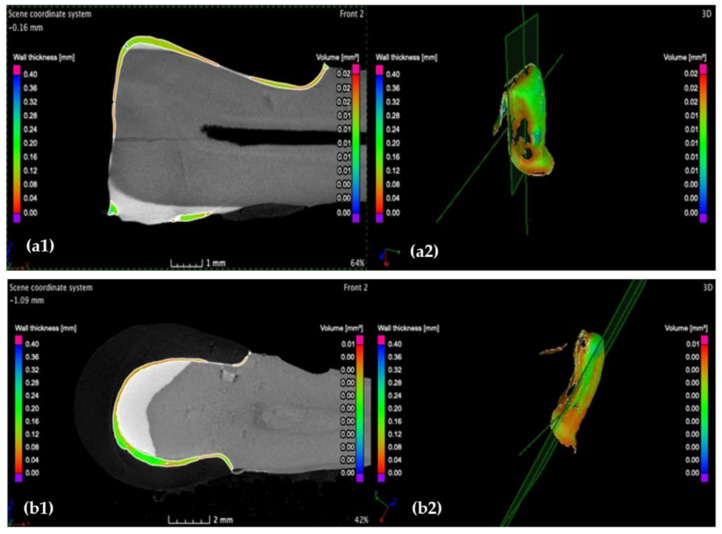
Investigation of the incisal marginal contour of (**a**) the CO and (**b**) CR tooth preparation in the frontal plane, displaying a maximum cement width *w* of 80 μm for both CO and CR samples. Notations: (**1**) section and (**2**) 3D reconstruction.

Further on, the samples are investigated in the frontal plane, moving the axis from the vestibular surface towards the tooth core (i.e., towards the pulp chamber), tracking the dimensional variations of the cement width, as shown in the example in Figure 11.

**Figure 12:**
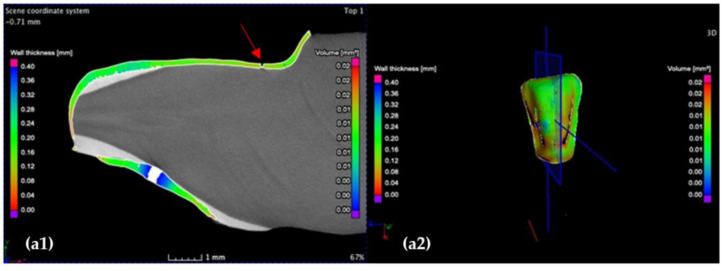

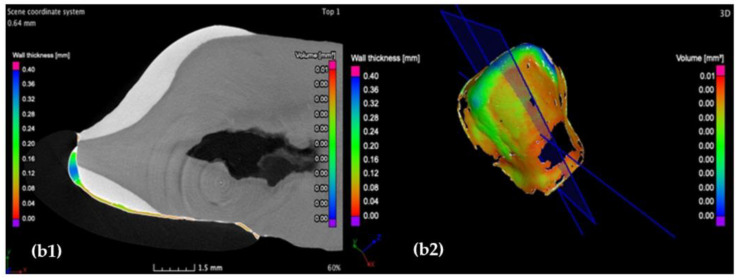
The highest cement thickness/width *w* situated in the middle of the vestibular face in sagittal section is 280 μm for (**a**) the CO and 100 μm for the CR (**b**) tooth preparation. Moreover, the layer of the luting cement of the CO sample features porosities within (red arrow) and is less uniform compared to the CR sample. Notations: (**1**) section and (**2**) 3D reconstruction.

The investigation in the sagittal plane is performed by moving the axis from the mesial to the distal surface, completing the 3D analysis of the wall width. An example from these investigations is shown in Figure 12.

Finally, the micro-CT investigations are completed, and the 3D analysis results are centralized and evaluated, as discussed in the following section. These investigations allow obtaining the graphs of the surface area with regard to the thickness/width of dental cement. Considering the simplest possible functions that can approximate these graphs on portions (i.e., parabolic or linear), they can be deduced following the methodology developed in [28,35].

### 2.5. Porosity Analysis

Porosity is a measure of the void spaces in a substance and is defined as the ratio of the volume of voids over the total volume [13]. Surface porosity is defined as the void spaces in the surface, which can increase the permeability of the set material. Additionally, the porosity analysis is important in identifying the localization of the voids included in the luting cement. As we use an auto-mixing luting cement syringe, the probability of voids inclusion by hand-malaxed methods is eliminated. Therefore, such an analysis only reveals the influence of the tooth preparation (CR versus CO) over the homogeneity of the cement. This is linked to tensions within adhesive interfaces that can lead to (numerous) fissures of the luting cement, further marginal percolation, dissolution, decays, and, eventually, debonding or fracture of the ceramic veneers.

**Figure 13:**
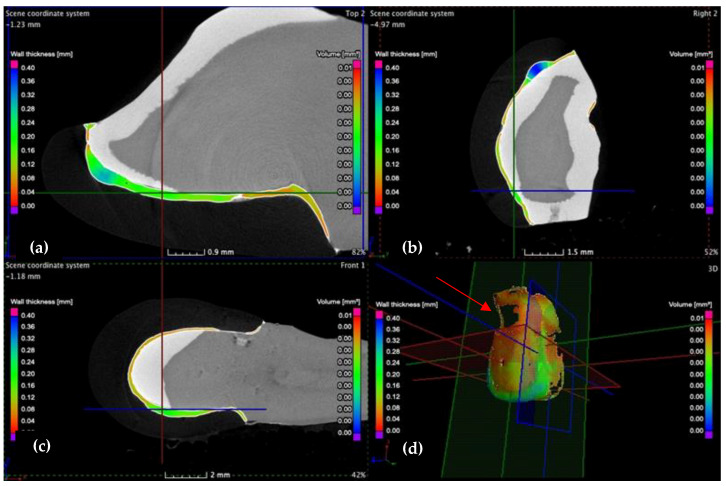
(**a**) Transversal, (**b**) frontal, and (**c**) sagittal sections, as well as (**d**) 3D scanning along the vestibular surface of the CR sample. Several small porosities are identified in the distal third. The lack of luting cement localized in the cervical third of the vestibular face (red arrow) suggests that the veneer is well-adapted to the tooth surface.

**Figure 14:**
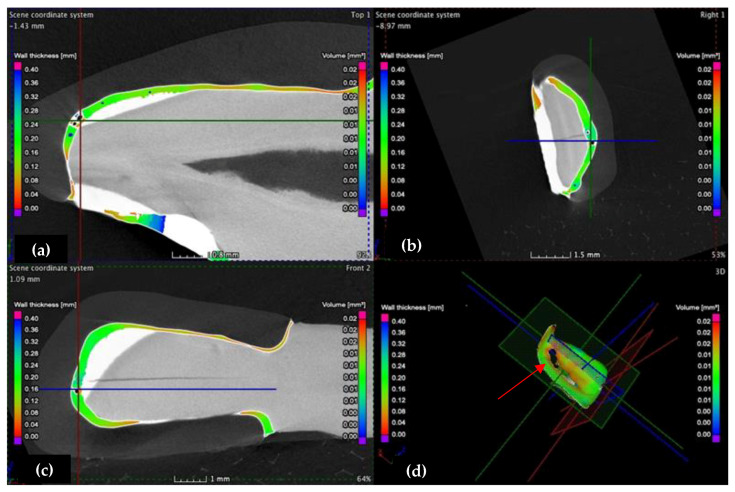
(**a**) Transversal, (**b**) frontal, and (**c**) sagittal sections, as well as (**d**) 3D scanning. This micro-CT investigation of the incisal adhesive interface of the CO sample reveals numerous porosities situated in the middle and mesial third of the incisal margin. The lack of luting cement (red arrow) suggests a good adaptation of the veneer to the tooth surface.

This analysis is also carried out using micro-CT, according to the three planes (X, Y, and Z), defined as transversal, frontal, and sagittal planes, respectively, in Figure 8. The investigation starts from the marginal interfaces (incisal, mesial, cervical, and distal) and continues along the internal adhesive interface. Two sections from these investigations are shown as examples in Figure 13 and Figure 14.

## 3. Results

### 3.1. Optical Microscopy

The marginal gaps of each of the 24 specimens are measured according to the points of interest described in Section 2.3. The results of all samples (presented as examples in Figure 3 and Figure 4) for the two groups of specimens are centralized in Supplemental Materials S1. Their statistical analysis is performed using the SPSS v.17 software, and the results are presented in Table 1. The differences between the two independent groups, CO and CR, are obtained by applying the Mann–Whitney U test, *p* < 0.001. It demonstrates that the average value of the marginal gap for the CO group is significantly higher than the average value for the CR group.

In conclusion, the novel CR veneers display a better marginal adaptation than CO. Moreover, compared to the clinically acceptable marginal adaptation of up to 120 μm [23,24,25], these results demonstrate that CR veneers may represent a more reliable prosthetic treatment option for in vivo oral rehabilitations.

### 3.2. Micro-CT

**Figure 15:**
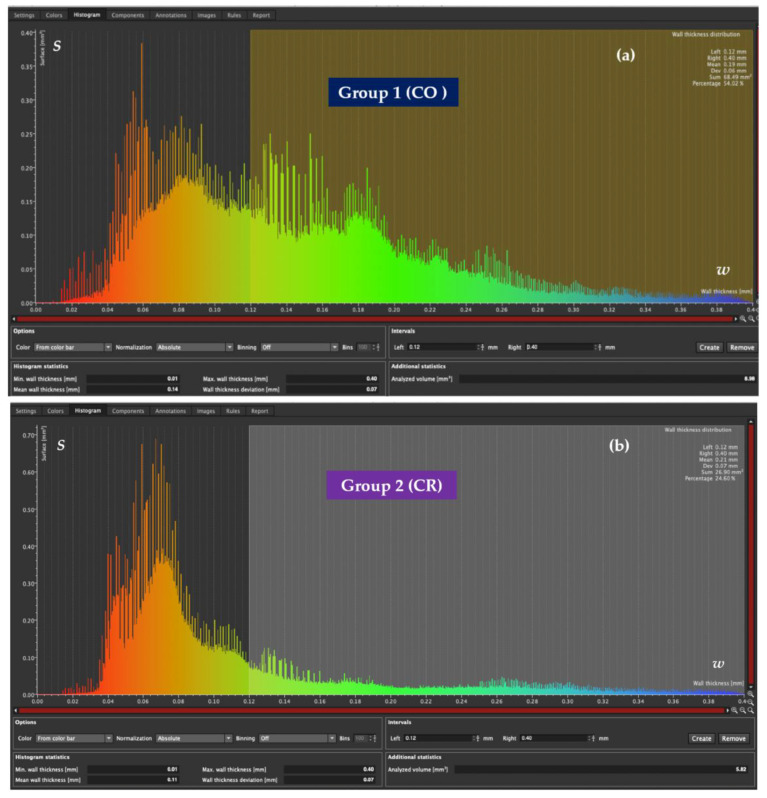
(**a**) Cement thickness/width *w* histogram of a CO tooth preparation, with the interval of *w* set from 0 to 120 μm. It reveals that 46.08% of the total preparation surface (of area *S*) is covered by this width interval, while 54.02% is covered by the second width interval, from 120 to 400 μm. (**b**) Cement width *w* histogram of a CR tooth preparation, with the interval of *w* set from 0 to 120 μm as well. The results show that 72.46% of the total preparation surface is covered by this width interval, while the 120 to 400 μm width interval covers only 26.40% of the total preparation surface.

Both wall thickness/width *w* and porosities analysis are used to elaborate a micro-CT report for each sample (Figure 15). The results concerning the marginal and internal adaptation of CO and CR veneers are presented in Supplemental Materials S2.

Throughout the analysis, the 120 μm criterion is considered as the maximum clinically acceptable cement width. The scan reports of the previously described samples (i.e., one from each group) are presented in the following.

To determine the proportion of this optimal width which covers the vestibular surface of the tooth preparation, the two relevant thickness/width intervals are chosen: up to 120 μm, and from 120 to 400 μm. The wall width histogram of each CO and CR sample (Figure 15) allows establishing the correlation between the width *w* (mm) of the luting cement and the corresponding covered surface area *S* (mm^2^). By comparing the two thickness/width histograms, the conclusion for the two considered examples is that the marginal and internal adaptation provided by the CR veneer is 63% higher than the CO one.

Considering all the 24 samples in the two groups, the results presented in Table 2 provide the average covered tooth area surface considering each width interval.

To compare the coverage percentage of the clinically acceptable cement width (0–120 μm) between the two experimental groups, the Mann–Whitney U Test, *p* < 0.001, is applied. Its results are presented in Table 3.

Based on the results obtained, we performed a posteriori sample size calculation, i.e., a Power test with the GPower 3.1.9.4 software, for T-test family with two tail(s), a “Laplace” Parent Distribution, 80% power, and 1 as an allocation ratio and effect size.

### 3.3. Mathematical Modeling of the Micro-CT Results

**Figure 16:**
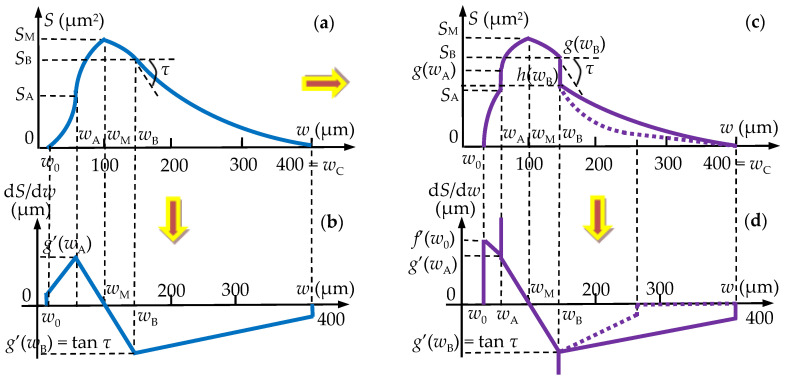
Mathematical modeling of the area *S* (μm^2^) of the luting cement surfaces as a function of its width *w* (μm), deduced for Group 1 (CO) from Figure 15a, as well as for Group 2 (CR) from Figure 15b. The initially considered *S*(*w*) functions and their gradients are presented in (**a**,**b**), respectively. The final *S*(*w*) functions and gradients deduced in Appendix A for both groups are represented in (**c**,**d**), respectively, with continuous line in the second part of the graph for Group 1 (CO) and with dotted line for Group 2 (CR).

Using the micro-CT diagrams in Figure 15, the graphs of the surface areas *S* of the luting cement can be obtained as functions of the width *w* (Figure 16a). Based on such a graph and considering the simplest equations (i.e., parabolic or linear) to approximate it on portions, the *S*(*w*) functions are deduced in Appendix A, with a methodology similar to the one developed in [31,32]. As discussed in detail in Appendix A, to simplify the modeling process (i.e., to use only second order and not higher-order polynomials), two vertical segments are introduced in the final graphs shown in Figure 16c.

These functions *S*(*w*), are provided, on specific width *w* intervals, in Table 4 and Table 5, for Group 1 (CO) and Group 2 (CR), respectively. The gradients of these functions are obtained as well—Figure 16d. Although the graphs have somewhat similar shapes for the CO and CR samples, the use of this gradient value for *w*_B_ (i.e., of the tangent *τ* of the function for *w = w*_B_) allows for a quantitative differentiation between the two graphs. On the other hand, to properly model analytically the diagrams obtained experimentally in Figure 15, one may observe that, for *w > w*_B_, a parabolic function is considered for Group 1 (CO), while a parabolic plus linear function must be considered for Group 2 (CR)—Figure 16c.

The functions deduced and presented in Table 4 and Table 5 also allow obtaining *the volume of luting cement in the interface*:

(1) $$V=\int_{0}^{w_{c}}S\left(w\right)\cdot dw\;\left[\mu m^{3}\right]$$

This synthetic parameter was deduced with this analytic method for the first time to our knowledge in [28]. It allows making a rigorous and simple comparison between the capability of the two different types of veneers to provide dental preparation as tight as possible. From this equation of the cement volume *V*, using the functions in Table 4 and Table 5, one obtains: Group 1 (CO), *V* = 42.2 × 10^6^ μm^3^; Group 2 (CR), *V* = 34 × 10^6^ μm^3^, respectively.

### 3.4. Porosity Results

**Figure 17:**
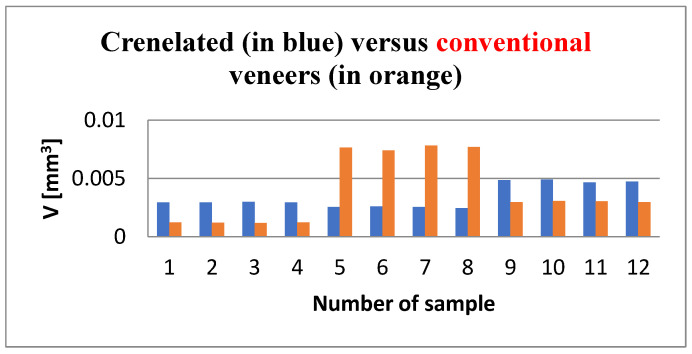
Comparison between the total volume of the voids/porosities (vertical axis) identified in the luting cement for each crenelated and conventional specimen (horizontal axis).

The *porosity analysis* triggered valuable data regarding the volume of the voids localized in the thickness of the luting cement in order to comparatively evaluate the influence of the tooth preparation type over the homogeneity of the adhesive layer. The volume of the porosities identified along the CR adhesive interfaces ranged from 2.4 × 10^6^ μm^3^ to 4.9 × 10^6^ μm^3^, whereas the CO adhesive interfaces displayed voids volumes ranging from 1.1 × 10^6^ μm^3^ to 7.8 × 10^6^ μm^3^. The porosity volumes of all 24 specimens are comparatively presented in Figure 17. To determine the differences between the two groups, the Mann–Whitney U Test was applied, and the results are presented in Table 6.

Analyzing these results, it can be concluded that the average for CR veneers is insignificantly low compared to CO ones, Mann–Whitney U Test, *p* = 0.755.

## 4. Discussion

Ceramic laminate restorations are one of the most successful treatment modalities for cosmetic improvement of unsightly anterior teeth. Treatment of traumatic fractures, moderate tooth wear, and congenital tooth malformations, as well as the aesthetic reshaping of anterior teeth, can be accomplished using such restorations. The long-term clinical performance of ceramic restoration depends on several factors, especially adaptation and relative strength. The marginal inaccuracy is another critical factor for an early failure of restorations [17,36,37]. Numerous experimental studies have been performed to evaluate the correlation between different materials used for indirect dental veneers and their marginal and internal adaptation to the tooth structure. Such studies have been mainly focused on crowns fabricated using conventional and digital workflows [38,39,40,41]. The influence of technological processes to elaborate indirect dental veneers on the marginal gap and internal discrepancies have also been intensively studied [19,42,43]. However, there are few studies that investigate the effect of different tooth preparations on the marginal internal accuracy of indirect dental veneers. Moreover, none of them describes the novel sinusoidal design, the “crenelated veneer” (CR), which we have proposed, to our knowledge, and is the subject of a patent of our group [26].

These CR veneers have been designed as a practical solution to augment the adhesive and mechanical properties of dental veneers. As demonstrated in the present study, they produce a smaller gap between tooth preparation and veneer; therefore, they can achieve higher retention due to the marginal micro-intrications between the dental structure and the veneer, as well as higher adhesion. Additionally, we observed that *they offer a more accurate positioning of the veneers* in situ *during the luting procedure*.

The importance of the marginal gaps and the internal discrepancies for the clinical success of ceramic restoration has been emphasized in several clinical trials [10,11]. However, various studies have shown controversial results concerning the clinically acceptable marginal gap values [12]. The criterion proposed by McLean and von Fraunhofer is to have marginal gaps of less than 120 μm for a successful restoration [13], and this has been widely accepted [14]. In this respect, regarding the manufacturing process of restorations, the CAD/CAM technology has the ability to machine high-strength ceramics (and titanium) with marginal gaps of less than 100 μm [15,16]. Therefore, this is the technique used in our study. Thus, we were able to comply with such marginal and internal discrepancies between 100 and 120 μm, in the range of clinical acceptance [23,24,25].

A limitation of the study is related to the fact that the considered teeth have different areas of luting surface and preparation geometry—in contrast to studies that proposed a standardized investigation by employing (identical) artificial teeth milled with CNC machines [44]. A way of reducing this latter issue is to consider the same types of teeth in both study groups, as described in Section 2.1.

The marginal and internal adaptation of the two considered groups of ceramic veneers (CO and CR) to natural tooth surfaces were investigated. The wall thickness/width analysis provided information regarding the width *w* of the luting cement localized underneath the ceramic veneer. This is equivalent to the internal discrepancy measured between the inner surface of the veneers and the vestibular face of the prepared teeth. The 120 μm criterion has been used as the maximum clinically acceptable cement width along the adhesive interface. Internal discrepancies larger than this limit can lead to cement shrinkage, microleakage, tensile forces beneath the veneers, and, eventually, to the debonding or fracture of the restorations.

The marginal gaps of the samples were investigated by using an optical digital microscope AmScope UWT. The measurements were performed along the peripheral circumference for all the veneer margins (mesial, distal, cervical, and incisal). The results represented the mean values of the gap measurements performed in five points for each segment of the investigated surface (Figure 3 and Figure 4). The CR veneers displayed better marginal adaptation than CO, as the mean value was 60 μm for the former and 230 μm for the latter (Table 1). Therefore, the novel design provides the premise to elaborate ceramic veneers with clinically acceptable margins. Precise marginal adaptation of an indirect dental restoration has always been crucial because cement is the weak restorative link: it is vulnerable to water absorption, polymerization shrinkage, wear, and microleakage; therefore, large marginal discrepancies can result in undue exposure of luting material to oral fluids [45]. Marginal gaps can also lead to secondary decays, tooth sensitivity, discolorations, and, as stressed above, fractures or debonding, eventually triggering the failure of the prosthetic treatment. Future work for such studies should include using Scanning Electron Microscopy (SEM) in the evaluation of marginal gaps [46].

The internal adaptation of the samples was evaluated using micro-CT. This particular aspect was correlated with the thickness/width w of the luting cement localized along the adhesive interface between the inner surface of the veneers and the vestibular surface of the samples. By performing the wall thickness analysis, the width w of the cement was divided into two intervals: 0–120 μm (i.e., clinically acceptable) and 120–400 μm (Figure 15). Statistical results showed that 81.5% of the total prepared tooth surface was covered by up to 120 μm cement thickness in the CR group, whereas the same cement thickness covered only 64.5% of the total prepared tooth surface of the CO group (Table 2 and Table 3). Cement film width w is a measure of the internal adaptation of the restoration. Not only a lower cement film width was observed for CR ceramic veneers, but a more even one as well. The latter indicates better seating compared to more irregular and thicker cement film widths observed for CO veneers. These findings are in agreement with May et al., who stated that the cement space should be uniform to facilitate seating without compromising retention or resistance forms [47]. A non-uniform cement layer can increase the maximum shear stresses in the cement at the bonding surface to values exceeding the bond strength of the cement; therefore, the cement layer should be as thin and uniform as possible [48].

Porosity analysis is important to assess the volume and distribution of voids accidentally included within the luting cement. Such voids can severely affect the physical and mechanical properties of the material. The material porosity may have an impact on numerous factors, including adsorption, permeability, strength, and density [49]. High porosity (altering the density, strength, and uniformity of the material) can eventually lead to leakages, micro-infiltration, and fissures within the cement layer. Moreover, the adhesive properties can be compromised, causing the debonding of veneers. According to the results obtained with a one-way ANOVA analysis, the following mean values of the porosities were 3.9 × 10^6^ μm^3^ for the CO and 3.4 × 10^6^ μm^3^ for the CR group (Table 6 and Figure 17). Even though the voids identified within the adhesive interface of the CR veneers were slightly smaller, there was no significant difference between the two experimental groups. Therefore, one may conclude that the homogeneity of the luting cement was not influenced by the tooth preparation design.

Coupling the above results with those obtained in a preliminary experimental study focused on mechanical properties of the novel design [27], but with veneers luted to resin tooth models (not to enamel), one may conclude that CR veneers can be superior to CO from a mechanical point of view as well: adhesive forces are increased by more than 60%, thus decreasing the probability of veneer detachment [27]; higher retention forces are produced due to the peripheral micro-retentions that form an intricated joint between the veneer and the substrate. Additionally, better contacts of the surfaces are provided, thus combining both adhesive and mechanical forces that prevent the veneer from debonding. Besides these, a more accurate in situ positioning of the veneers during the luting procedure is made possible. Therefore, this in vitro research demonstrated that CR veneers might represent a successful long-term treatment option in aesthetic dentistry. Their longevity (which is subject of future work in our group) may be based on their sinusoidal contour and peripheral intrications with the substrate.

From the calculus of the cement volume *V*, performed using the functions deduced in Table 4 and Table 5, one obtains: Group 1 (CO), *V* = 42.2 × 10^6^ μm^3^; Group 2 (CR), *V* = 34 × 10^6^ μm^3^. The approximate 20% reduction in volume from the CO to the CR sample demonstrates the performance of the novel veneer.

This confirms the results obtained from comparing statistically not only a sample from each group but all the 12 preparations in each group from the point of view of the covered surface (for a width *w* smaller than the clinically acceptable threshold of 120 μm) in Table 2 and Table 3. Thus, the two approaches may validate each other, although the volume calculus is more accurate. On the other hand, the covered surface analysis is more suggestive and relevant from the point of view of the adhesion.

This complementary methodology can be applied for micro-CT studies of (different) dental materials, but other imaging techniques, such as Optical Coherence Tomography (OCT) [50,51], can benefit from such a quantitative assessment as well. Such approaches are subject of future work, as OCT has the advantage to provide real-time, in vivo, and non-invasive investigations in the oral cavity (with higher resolution than radiography [52,53]), eventually using dedicated handheld scanning probes [54,55].

## 5. Conclusions

Based on the findings of this in vitro study, the following conclusions were drawn regarding the novel patented veneers design [26]:

(i) Crenelated (CR) ceramic dental veneers, with their marginal sinusoidal contour, displayed a higher marginal adaptation (60 μm) than the conventional (CO) (with linear margins) veneers (230 μm).
(ii) The internal adaptation was considerably better for the CR group in comparison with CO, as the clinically accepted cement thickness/width of up to 120 μm covered 81.5% of the tooth surface for CR compared to 64.5% for CO. Such a thinner and more uniform layer of luting cement creates the premises for better protection of restorations from cement shrinkage, microleakage, tensile forces beneath the veneers and, eventually, debonding or fracture-aspects to be investigated in future work.
(iii) The homogeneity of the luting cement was similar for CR and CO veneers.
(iv) Micro-CT proved to be reliable and precise to evaluate the internal adaptation of the restorations, as well as the porosities localized in the luting cement. It has the advantage of providing 3D information on the entire volume of dental cement (i.e., the interface between dental support and veneers). The more common and accessible tool, optical microscopy, provided valuable data regarding the marginal adaptation, with a good agreement with the micro-CT results, but cannot evaluate internal adaptation.

In conclusion, the first study hypothesis was verified: the novel CR veneers provide a better marginal and internal adaptation. However, the second study hypothesis was not verified, as both groups have a similar homogeneity of the dental cement, as concluded from the porosity analysis.

In addition, the peripheral micro-retentions that form an intricated joint between the veneers and the substrate are deemed to provide higher retention and adhesive forces, more accurate positioning of the veneers in situ during the luting procedure, and a more appropriate color adaptation along the adhesive interfaces. As CR dental veneers produced a more homogenous and thinner dental cement film, they can improve the resistance to microleakage compared to CO veneers. All these aspects are subject of future work. Additionally, further in vivo studies are considered to assess such clinical performances of the novel CR veneers. The long-term survival and complication rates are of special interest [56]. Following our preliminary study where the adhesive forces between the novel veneers and resin tooth models were investigated [27], future work must also address crenelated veneers adhesively luted on natural human teeth. As the cement thickness is related to microleakage, both debonding and microleakage must be investigated, as well. Finally, as with today’s dentistry tools, manufacturing the novel, crenelated veneers may be a delicate process, we are approaching new, more efficient methods for obtaining such veneers.

## Figures and Tables

**Table 1:** Comparison between the statistical results triggered by the gap measurement/width *w* (μm) analysis of CR versus CO veneers.

Type of Sample	*N*	Mean width *w* (μm)	Standard Deviation (μm)	Standard Error Mean (μm)
Conventional (CO)	12	235.9	89.38	7.45
Crenelated (CR)	12	64.3	26.25	2.19

**Table 2:** Average covered surface of the two groups for different intervals of cement width.

Intervals of Cement width *w* (μm)	Average Covered Surface *S* (%)	
	Group 1, CO Veneers	Group 2, CR Veneers
0–120	64.55	81.56
120–400	35.45	18.31

**Table 3:** Descriptive comparison of the covered surface *S* (%) for the clinically acceptable threshold.

Group	*N*	Mean ± Standard Deviation (%)	Standard Error Mean (%)	Mean Rank	Sum of Ranks	p
(1) CO veneers	12	64.56 ± 9.728	2.808	6.92	83.00	<0.001 ^s^
(2) CR veneers	12	81.57 ± 4.619	1.333	18.08	217.00	

Legend: ^s^—significant difference.

**Table 4:** Modeling of the functions of surface area *S* versus the width *w* of the luting cement for the Group 1 (CO) sample.

Group	Parameters *w* (μm); *S* (10^3^ μm^2^)	Area *S* (μm^2^) of the Surface of Adhesive Layer with a Certain width *w* (μm)		
		*w*_0_ < *w* < *w*_A_	*w*_A_ < *w* < *w*_B_	*w*_B_ < *w* < *w*_C_ = *w*_max_
1 (CO)	*w*_0_ = 30; *S*_0_ = 0	*f*(*w*) = (*w* − *w*_0_){*τ*(*w*_A_ − *w*_M_)(*w* − *w*_A_)/(*w*_B_ − *w*_M_) − *S*_A_ [*w* − (2*w*_A_ − *w*_0_)]/(*w*_A_ − *w*_0_)}/(*w*_A_ − *w*_0_) *f*(*w*) = −62.47 · *w*^2^ + 62,499 · *w* − 56,239	*g*(*w*) = *S*_M_*+ τ*· (*w* − *w*_M_)^2^/[2(*w*_B_ − *w*_M_)] *g*(*w*) = −14.43 · *w*^2^ + 2.31 · *w* − 179,815.03	*h*(*w*) = (*w* − *w*_C_){*τ*(*w* − *w*_B_) − *S*_B_ · [*w* + (*w*_C_ − 2*w*_B_)]/(*w*_B_ − *w*_C_)}/(*w*_B_ − *w*_C_) *h*(*w*) = 1.78 · *w*^2^ + 356.52 · *w* + 142,299.3
	*w*_A_ = 40; *S*_A_ = 180			
	*w*_M_ = 60; *S*_M_ = 360			
	*w*_B_ = 80; *S*_B_ = 300			
	*w*_C_ = 400 = *w*_max_; *S*_3_ = 0			

**Table 5:** Modeling of the functions of surface area *S* versus the width *w* of the luting cement for the Group 2 (CR) sample.

Group	Parameters *w* (μm); *S* (10^3^ μm^2^)	Area *S* (μm^2^) of the Surface of Adhesive Layer with a Certain Width *w* (μm)			
		*w*_0_ < *w* < *w*_A_	*w*_A_ < *w* < *w*_B_	*w*_B_ < *w* < *w*_P_	*w*_B_ < *w* < *w*_C_ = *w*_max_
2 (CR)	*w*_0_ = 10; *S*_0_ = 0	The same expression for *f*(*w*) as in Table 4 *f*(*w*) = −1999.9 · *w*^2^ + 143,987.9 · *w* − 899,930.7	The same expression for *g*(*w*) as in Table 4 *g*(*w*) = −0.043 · *w*^2^ + 5.19 · *w* + 358,441.15	*h*(*w*) = (*w* − *w*_P_){*τ*(*w* − *w*_B_) − (*S*_B_ − *S*_P_) · [*w* + (*w*_P_ − 2*w*_B_)]/(*w*_B_ − *w*_P_)}/(*w*_B_ − *w*_P_) *h*(*w*) = 53.06 · *w*^2^ − 594,687.08 · *w* + 79,294.92	*j*(*w*) = *S*_P_ · (−*w + w*_C_)/(*w*_C_ − *w*_P_) *j*(*w*) = −160 · *w* + 64,000
	*w*_A_ = 50; *S*_A_ = 100				
	*w*_M_ = 80; *S*_M_ = 180				
	*w*_B_ = 100; *S*_B_ = 160				
	*w*_P_ = 150; *S*_P_ = 40				
	*w*_C_ = 400 = *w*_max_; *S*_3_ = 0				

**Table 6:** Statistical comparison of the porosity volume *V*_p_ (10^6^ μm^3^) between CR and CO veneers.

Group	*N*	Mean (10^6^ μm^3^)	Standard Deviation (10^6^ μm^3^)	Min. (10^6^ μm^3^)	Max. (10^6^ μm^3^)	Mean Rank	Sum of Ranks
(1) CO veneers	12	3.42276	1.022662	2.442	4.906	12.00	144.00
(2) CR veneers	12	3.94780	2.833850	1.175	7.829	13.00	156.00

## Data Availability

The data presented in this study are available within the article and in Supplementary Materials S1 and S2.

## Supplementary Materials

The following are available online at https://www.mdpi.com/article/10.3390/medicina57080772/s1.

## Appendix A

### Areas S (μm^2^) of the Luting Cement Surfaces as Functions of Their Widths w (μm)

For each of the three portions that can be identified in the *S*(*w*) graphs in Figure 16, we consider a parabolic function. These functions must be deduced considering the parameters pointed out for each considered sample (from Group 1 and 2, respectively) in Table 4 and Table 5.

For the first portion of *S*(*w*), for the interval *w*_0_ < *w* < *w*_A_, we consider the function

*f*(*w*) = *aw*^2^ + *bw* + *c*, with *f′*(*w*) = 2*aw* + *b*, where *a*, *b*, *c* = cst. (A1)

To obtain the constants *a*, *b,* and *c* we impose from Figure 16 the conditions:

*f*(*w*_0_) = 0;  *f*(*w*_A_) = *S*_1_;  *f*’(*w*_A_) = *g′*(*w*_A_), (A2)

but to apply the third condition, the value of the derivative for *w* = *w*_A_ must be first obtained from the second portion of the *S*(*w*) function.

For this second portion of *S*(*w*), for the interval *w*_A_ < *w* < *w*_B_, another parabolic function is considered:

*g*(*w*) = *dw*^2^ + *ew* + *k*, with *g′*(*w*) = 2*dw* + *e*, where *d*, *e*, *k* = cst. (A3)

From Figure 16 the following conditions are imposed:

*G′*(*w*_M_) = 0;  *g*(*w*_M_) = *S*_M_;  *g′*(*w*_B_) = *τ*, (A4)

where *τ* is the value of the angle made by the tangent to the graph with the horizontal for *w* = *w*_B_. The value of this angle is obtained from Figure 15 for each considered sample: it is equal to approximately π/6 (rad) for the CO sample—Figure 15a, and to approximately π/3 (rad) for the CR sample—Figure 15b. This angle, therefore, the third condition in Equation (A4), is considered instead of a condition *g*(*w*_B_) = *S*_2_. This allows for more precise modeling of the shape of the diagram in Figure 16. The value of *g*(*w*_B_) remains to be calculated, and it could be a verifying condition for the accuracy of the performed modeling.

With Equation (A4) in (A3), the coefficients of the second parabolic function are:

*d* = *τ*/2(*w*_B_ − *w*_M_); *e* = − *τ*· *w*_M_/2(*w*_B_ − *w*_M_); *k* = *S*_M_*+ τ*· *w*_M_^2^/2(*w*_B_ − *w*_M_). (A5)

Using the parameters in Table 5 and Table 6, we obtain from Equation (A5) the coefficients *d*, *e,* and *k* for both groups: for Group 1, *d*_1_ = −14.434, *e*_1_ = 2.309 μm, and *k*_1_ = 179,815.03 μm^2^; for Group 2, *d*_2_ = −0.043, *e*_2_ = 5.196 μm, and *k*_2_ = 358,441.15 μm^2^.

From the *g*(*w*) function obtained with the coefficients given by Equation (A5), its derivative in *w* = *w*_A_ is

*g′*(*w*_A_) = *τ*·(*w*_A_ − *w*_M_)/(*w*_B_ − *w*_M_) = *τ*_A_ (A6)

Thus, returning to the first portion *f*(*w*), using Equation (A6) in Equations (A1) and (A2), the coefficients of this first function are obtained:

*a* = [*τ*_A_ − *S*_A/_(*w*_A_ − *w*_0_)]/(*w*_A_ − *w*_0_); *b* = [−*τ*_A_ (*w*_A_ + *w*_0_) + 2·*S*_A_·*w*_A/_(*w*_A_ − *w*_0_)]/(*w*_A_ − *w*_0_); *c* = *w*_0_ [*τ*_A_·*w*_A_ − *S*_A_·(2*w*_A_ − *w*_0_)/(*w*_A_ − *w*_0_)]/(*w*_A_ − *w*_0_) (A7)

Therefore, using the parameters in Table 4 and Table 5, one obtains from Equation (A7) the coefficients *a*, *b,* and *c*: for Group 1, *a*_1_ = −62.47, *b*_1_ = 62,499 μm, and *c*_1_ = −56,239 μm^2^, while for Group 2, *a*_2_ = −1999.9, *b*_2_ = 143,987.9 μm, and *c*_2_ = −899,930.7 μm^2^.

For the third portion of *S*(*x*), i.e., for the interval *w*_B_ < *w* < *w*_C_ = *w*_max_, another parabolic function, *h*(*w*) is considered for Group 1 (CO):

*h*(*w*) = *mw*^2^ + *nw* + *r*, with *g*’(*w*) = 2*mw* + *n*, where *m*, *n*, *r* = cst. (A8)

From Figure 16, the following conditions are extracted:

*h′*(*w*_B_) = *τ*;  *h*(*w*_B_) = *S*_B_; ; *h*(*w*_C_) = 0. (A9)

Using Equations (A9) in (A8), the coefficients of this function are obtained:

*m* = [*τ* − *S*_B_*/*(*w*_B_ − *w*_C_)]/(*w*_B_ − *w*_C_); *n* = [−*τ*· (*w*_B_ + *w*_C_) + 2*w*_B_ · *S*_B_*/*(*w*_B_ − *w*_C_)]/(*w*_B_ − *w*_C_); *r* = *w*_C_ · [*τ*· *w*_B_ + *S*_B_ · (*w*_C_ − 2*w*_B_)/(*w*_B_ − *w*_C_)]/(*w*_B_ − *w*_C_). (A10)

Using the parameters in Table 4, from Equation (A10) the coefficients for Group 1 are: *m*_1_ = 1.78, *n*_1_ = 356.52 μm, and *r*_1_ = 142,299.3 μm^2^.

For Group 2 (CR), the third portion of *S*(*x*), i.e., the parabolic function, *h*(*w*) with Equation (A8) is considered, but only for the interval *w*_B_ < *w* < *w*_P_,. From Figure 16, the necessary conditions to obtain the coefficients of the function are:

*H′*(*w*_B_) = *τ*;  *h*(*w*_P_) = *S*_P_;  *h*(*w*_B_) = *S*_B_. (A11)

Using Equations (A11) in (A8), the coefficients of this function are obtained:

*m* = [*τ −* (*S*_B_*− S*_P_)*/*(*w*_B_ − *w*_P_)]/(*w*_B_ − *w*_P_); *n* = [−*τ*· (*w*_B_ + *w*_P_) + 2*w*_B_ · (*S*_B_ − *S*_P_)/(*w*_B_ − *w*_P_)]/(*w*_B_ − *w*_P_); *r* = *w*_P_ · [*τ*· *w*_B_ + (*S*_B_*− S*_P_) · (*w*_P_ − 2*w*_B_)/(*w*_B_ − *w*_P_)]/(*w*_B_ − *w*_P_). (A12)

Using the parameters in Table 5, from Equation (A12) the coefficients for Group 2 are: *m*_2_ = 53.06, *n*_2_ = 594,687.08 μm, and *r*_2_ = 79,294.92 μm^2^.

Additionally, for Group 2 (CR), a fourth portion of *S*(*x*) must be considered, i.e., for the interval *w*_P_ < *w* < *w*_C_. This is a linear function

*j*(*w*) = *qw* + *z*, where *q*, *z* = cst. (A13)

From Figure 16, the conditions to obtain its coefficients are

*j*(*w*_P_) = *S*_P_;  *j*(*w*_C_) = 0. (A14)

Using the parameters in Table 5, from Equation (A14), these coefficients are (for Group 2), *q* = 160 μm, and *z* = 64,000 μm^2^.

From Equations (A3) and (A5), the following conditions are obtained:

*g*(*w*_A_) = *τ*· (*w*_A_ − *w*_M_)^2^/2(*w*_B_ − *w*_M_) + *S*_M_*;  g*(*w*_B_) = *τ*· (*w*_B_ − *w*_M_)/2 + *S*_M_ (A15)

Using the values of the parameters in Table 4, from Equation (A15), for Group 1, *g*(*w*_A_) = 179,774 μm^2^, while in Table 4, it was considered *S*_A1_ = *f*(*w*_A_) = 100,000 μm^2^. Using the values of the parameters in Table 5, for Group 2, *g*(*w*_A_) = 359,978 μm^2^, while in Table 5, it was considered *S*_A2_ = *f*(*w*_A_) = 180,000 μm^2^.

Using the values provided in Table 4, from Equation (A12), for Group 1, *g*(*w*_B_) = 179,994 μm^2^, while it was considered in Table 4 that *S*_B1_ = *h*(*w*_B_) = 160,000 μm^2^. From Table 5, for Group 2, *g*(*w*_B_) = 359,983 μm^2^, higher than the considered value in Table 5, *S*_B2_ = *h*(*w*_B_) = 300,000 μm^2^.

However, as the values calculated from Equation (A15) refer to the second function *g*(*w*), while the values considered in Table 4 and Table 5 were considered to deduce the first function *f*(*w*) and the third one *h*(*w*), we can introduce a vertical segment to close the gap between the end of the graph of *f*(*w*) and the beginning of the graph of *g*(*w*), for *w* = *w*_A_, as well as between the end of the graph of *g*(*w*) and the beginning of the graph of *h*(*w*), for *w* = *w*_B_. This final, correct modeling is shown in Figure 16c,d, in contrast to the initially considered modeling shown in Figure 16a,b. This procedure keeps the modeling process simple; otherwise, much more complicated functions than second-order polynomials should have been considered.

The deduced functions *f*(*w*), *g*(*w*), and *h*(*w*) of the *S*(*w*) graph are provided in Table 4 for Group 1. The deduced functions *f*(*w*), *g*(*w*), *h*(*w*), and *j*(*w*) of the *S*(*w*) graph are provided in Table 5 for Group 2. As a remark, the values of the functions’ gradients for *w* = *w*_A_ and *w* = *w*_B_ tend to infinity because of the considered vertical segments in these points. Additionally, one can demonstrate that, for the given values, *f’*(*w*_A_) > *g’*(*w*_A_) for both samples, hence the final shape of the gradients’ graphs in Figure 16d.
